# Monoclonal antibody specific to HA2 glycopeptide protects mice from H3N2 influenza virus infection

**DOI:** 10.1186/s13567-015-0146-7

**Published:** 2015-03-19

**Authors:** Xing Xie, Yan Lin, Maoda Pang, Yanbing Zhao, Dildar Hussain Kalhoro, Chengping Lu, Yongjie Liu

**Affiliations:** College of Veterinary Medicine, Nanjing Agricultural University, Nanjing, China

## Abstract

Canine influenza virus (CIV) subtype H3N2 is a newly identified, highly contagious respiratory pathogen that causes cough, pneumonia and other respiratory symptoms in dogs. Data indicate that the virus is responsible for recent clinical cases of dog disease in China. However, therapeutic options for this disease are very limited. In this study, seven monoclonal antibodies (mAbs) against CIV JS/10 (an H3N2 subtype virus) were produced and characterized. Among them, mAb D7, which is specific for the HA2 glycopeptide (gp), induced the highest neutralization titers. The protection provided by mAb D7 was evaluated in BALB/c mice challenged with homologous or heterologous strains of H3N2 influenza virus, including two strains of CIV and one strain of swine influenza virus (SIV). The data show that mAb D7 protected the mice from infection with the three viral strains, especially the homologous strain, which was indicated by the recovery of body weight, reduction of viral load, and reduction of tissue damage. Moreover, the levels of IFN-γ and TNF-α in the lungs, as detected by ELISA, were reduced in the infected mice treated with the mAb D7 compared with those without mAb D7 treatment. Thus, our findings demonstrate, for the first time, that a mAb could reduce the release of IFN-γ and TNF-α associated with tissue damage by CIV infection and that the mAb might be of great therapeutic value for CIV infection.

**Electronic supplementary material:**

The online version of this article (doi:10.1186/s13567-015-0146-7) contains supplementary material, which is available to authorized users.

## Introduction

Influenza A virus, a highly contagious pathogen, can infect both birds and mammals. It has undergone significant genetic variation to adapt to different hosts [1]. Its interspecific transmission is achieved by the recombination or direct transfer of genetic material [2]. The first case of dog infection with H3N8 canine influenza virus (CIV) was reported in the USA in 2004 [3,4], followed by a report of CIV in South Korea, which subsequently demonstrated that CIV was able to transmit directly from dog to dog [5,6]. Recently, the first case of H3N2 CIV infection was reported in Guangdong Province in 2010 [7]. Over recent years, infection with H3N2 CIV in dogs has developed from scattered cases to wide distribution across the country [8-10]. Dogs have no natural immunity to this virus, thus a number of preventive and therapeutic measures against CIV have been attempted to control the prevalence of this virus. Among them, vaccination is an important method to prevent and control influenza virus infection [11-13]. Current vaccine research against CIV has made some progress. In 2009, the U.S. Department of Agriculture (USDA) approved a list of vaccines against H3N8 CIV, which could effectively reduce viral shedding [14]. In 2012, the patent for an H3N2 CIV vaccine in South Korea was also approved [15]. Preventive vaccination is historically the primary measure to control influenza virus infection, but it has some limitations [16]. For example, influenza vaccines may not be effective enough to prevent against divergent viral strains, or may be less immunogenic and effective in certain groups, such as the very young, the old, and the immunocompromised [17]. Therefore, it is crucial to develop other measures to protect animals from infection/disease [18]. For example, passive immunity by transferring a specific antibody to a recipient could protect animals from infection [19]. Monoclonal antibodies (mAbs) can neutralize viruses, thus preventing virus attachment to, or fusion with, the host cell [20]. Many studies have demonstrated that mAbs are an effective and preventive treatment against human-origin [21-23] or avian-origin influenza virus infection [11,24,25]. However, to date, there are no neutralizing mAbs available to prevent and control H3N2 CIV infection.

In this study, we identified seven mAbs against H3N2 CIV, and tested one of them, the D7 mAb, against three different H3N2 subtype virus strains in animal experiments. This is the first description of a neutralizing mAb against H3N2 CIV.

## Materials and methods

### Virus strains, cells and medium

Three viral strains of the H3N2 subtype, including A/Canine/Jiangsu/06/2010 (JS/10), A/Canine/Guangdong/12/2012 (GD/12) and A/swine/Shandong/3/2005 (SD/05) were used in this study. The GenBank accession numbers of JS/10, GD/12 and SD/05 are JN247616 to JN247623, KF826944 to KF826951 and EU116037 to EU116044, respectively. The three viral strains were adapted to mice by passaging 3 times. They were propagated in 10-day-old specific-pathogen free (SPF) embryonated chicken eggs and stored at −70 °C before use.

Madin-Darby canine kidney (MDCK) cells were cultured in Dulbecco’s modified essential medium (DMEM) containing 10% (v/v) fetal bovine serum (Hyclone, tah, USA) and maintained at 37 °C and in a 5% (v/v) CO_2_ atmosphere.

### Experimental animals

BALB/c mice (6 weeks old, female) were purchased from the Animal Experiment Center, Yangzhou University. All animal experiments complied with the guidelines of the Animal Welfare Council of China, and the Animal Ethics Committee of Nanjing Agricultural University approved the study.

### Fifty-percent tissue culture infective dose (TCID_50_) assays

One day before infection, a 96-well dish containing a monolayer of MDCK cells was prepared. The next day, serial dilutions of the three influenza virus strains were made, and the cell monolayers were laterally inoculated; each dilution had three replicates. The cytopathic effect (CPE) was observed daily and the numbers of wells for a virus dilution that showed more than and less than 50% pathological changes were recorded. TCID_50_ titers were calculated in accordance with the Reed-Muench method [26].

### Generation of H3N2 mAbs

Canine influenza virus JS/10 was grown in 10-day-old SPF embryonated chicken eggs at 37 °C for 72 h. Allantoic fluids were harvested and the hemagglutination (HA) activity of the allantoic fluids was tested at room temperature using 1% chicken red blood cells (RBC). HA titers more than or equal to 1:64 were selected, and the virus was purified using differential centrifugation and sucrose density gradient centrifugation. Preparation of anti-H3 mAbs followed standard hybridoma technology, as previously described [27]. Six-week-old female BALB/c mice were injected intracutaneously with 100 μg of purified virus JS/10 using complete Freund’s adjuvant (Sigma, Beijing, China) as the primary adjuvant, followed by incomplete Freund’s adjuvant. Three days before harvesting the splenocytes, 100 μg of JS/10 were inoculated intravenously. Isolation and screening of the hybridomas was performed as described previously [28]. MAbs were prepared by injecting hybridoma cells into the peritoneal cavities of pristane-primed BALB/c mice. The ascetic fluid was collected after 9–12 days and inactivated at 56 °C for 30 min.

### Hemagglutination inhibition (HI) and microneutralization tests

The HI test was performed to assess antibody reactivity against three H3N2 strains, JS/10, GD/12 and SD/05, as previously described [29]. Briefly, 25 μL of serial two-fold dilutions of the purified 5-fold diluted ascetic fluid of the mAb were mixed with 4 HA units of virus in disposable hemagglutination plates and incubated at 37 °C for 30 min. Then, 25 μL of 1% chicken RBC were added to each well and incubated at room temperature for 30 min. To rule out non-specific inhibition, in the HI assay, we used the ascetic fluid produced with the injection of SP2/0 myeloma cells as a negative control. The HI titer was expressed as the reciprocal of the highest serum dilution that completely inhibited hemagglutination of 4 HA units of the virus [30].

Cell-based neutralization assays were performed as previously described [31]. A dose of 100 TCID_50_ of viruses was used in the assays. Supernatants of ascites were tested at a starting dilution of 1:25. Briefly, two-fold dilutions of hybridoma supernatants were mixed with virus suspension containing 100 TCID_50_ of purified H3N2 virus and incubated at 37 °C in a 5% CO_2_ incubator for 1 h before their addition to a monolayer of MDCK cells in 96-well plates. One hundred microliters of serum-free DMEM was added to each well and incubation at 37 °C continued for 1 h. The cytopathic effect was observed every 24 h for 48 to 72 h.

### Antigen identification of MAbs

To determine the recognized HA domain of the MAbs, we recombinantly expressed the HA, HA1 and HA2 proteins of virus JS/10. The recombinant proteins were subjected to SDS-PAGE under reducing conditions. The proteins were then electro-transferred and immobilized on a nitrocellulose membrane. The membrane was blocked with 5% nonfat milk in phosphate buffered saline (PBS) containing 0.1% Tween 20 (PBST) at 37 °C for 1 h. The membrane was subsequently incubated with the mAb prepared in this study, rinsed in PBST, and incubated with horseradish peroxidase (HRP)-conjugated rabbit anti-mouse immunoglobulin (Bio-Rad, Shanghai, China), followed by incubation with chromogenic reagents (Tiangen, Beijing, China) [32].

### Cross-protection by H3-specific mAb

BALB/c mice were used to determine the protective efficacy of mAb D7. Intranasal inoculations with 10^7^ TCID_50_ of virus strains JS/10, GD/12 and SD/05 were given to experimental groups I (*n* = 45), II (*n* = 45) and III (*n* = 45), respectively; the control group received PBS (*n* = 15). Each experimental group was divided into three subgroups (*n* = 15 for each subgroup), which were the virus-infected, mAb D7 and irrelevant mAb IgG subgroups, respectively. Mice injected with PBS or irrelevant mAb IgG were considered as blank and negative controls, respectively. For the mAb D7 and irrelevant mAb IgG subgroups, mice were pretreated intraperitoneally with mAb D7 (36 μg/mL) or irrelevant mAb IgG (32 μg/mL) against IgM from Chinese breams developed in our laboratory, at a dose of 20 mg per kg of body weight in 100 μL of PBS before the viral challenge [24,33,34]. After 24 h, mice were challenged with three different H3N2 strains. Mice were observed daily to monitor body weight and clinical symptoms for up to 14 days.

Three mice from each subgroup were euthanized humanely according to a pre-designated schedule. At 2, 4, 6, 10 and 14 days post-infection (dpi), blood samples and tissues including heart, spleen, lung, brain and intestine, as well as feces were collected. Virus shedding was detected by screening fecal samples. Detection of viral RNA was used to determine tissue distribution and virus shedding. Tissues and feces were homogenized in lysates at a ratio of 1:1 (g/mL), respectively, centrifuged at 10 000 × *g* for 30 min, and the supernatants were collected for the extraction of viral RNA using the Virus Nucleic Acid Extraction Kit II (Geneaid, Taiwan). All tissues collected above, including blood, were used for virus titration; the lung, brain and heart were also used for histological and immunohistochemical analysis at 6 dpi.

### Real-time PCR for quantitation of viral loads

Quantitative assays were carried out to measure viral loads in the blood and main organs [33]. Total RNA from tissues and blood samples was reverse transcribed using the PrimeScript™ RT reagent Kit with gDNA Eraser (Perfect Real Time) (Takara, Dalian, China) and then run on an ABI 7500 Real Time PCR System using the SYBR1 Premix Ex TaqTM (Perfect Real Time) kit (Takara). Reverse transcription and cDNA amplification were carried out as described previously [8]. The primers used were designed against a region of the matrix gene: 5′- TCTATCGTCCCATCAGGC/GGTCTTGTCTTTAGCCATTC-3′. A reference standard was prepared using pMD19-T Simple Vector (50 ng/μL; Takara) that contained the corresponding target virus sequences. A series of eight 10-fold dilutions equivalent to 1 × 10^3^–1 × 10^10^ copies per reaction were prepared to generate calibration curves and were run in parallel with the test samples [35]. RNA of the amount of the two avian-origin CIV and human-origin influenza viruses was calculated from the standard curve by real-time RT-PCR. The detection limit of this assay was 1120 copies of RNA per mL.

### Histopathology and immunohistochemical analysis

After euthanasia at 6 dpi, the heart, brain and lung from the mice inoculated with JS/10, GD/12, SD/05 or PBS were collected and placed into 10% neutral buffered formalin. After fixation the tissues were embedded in paraffin, sectioned at 4 μm and stained with hematoxylin and eosin for histological evaluation. Sequential slides were stained using an immunoperoxidase method [8]. Expression of hemagglutinin in tissues was examined by immunohistochemical staining of histological sections. In brief, sections were blocked with 1% bovine serum albumin/PBS, stained with mAb D7 at a dilution of 1:5000 for one hour at 37 °C, followed by biotin conjugated goat anti-mouse immunoglobulin (Bio-Rad) at a dilution of 1:200 for 30 min at 37 °C. The sections were subsequently incubated with HRP conjugated streptavidin (Bio-Rad) at 37 °C for 30 min. Sections were then developed with HRP-DAB chromogenic substrate kit (Tiangen) for 10 min and counterstained with hematoxylin. The lungs were assigned a grade of 0 to 3 based on the histological character of the lesions. Score criteria of different grades were in accordance with a previous study [36].

### Measurement of cytokines

We further investigated if there was a correlation between severe disease and inflammatory cytokine production in virus-challenged mice, and ascertained whether passive immunization with antibodies affected the levels of cytokines involved in defense against three different influenza virus infections. Sections of the lungs (alternating right and left lungs) from all the mice were homogenized in 1 mL of PBS per 1 g of lung tissue. The homogenates were centrifuged, and the supernatants were frozen at −70 °C until tested. The supernatants were assayed for gamma interferon (IFN-γ) and tumor necrosis factor (TNF-α) using ELISA kits (Sigma-Aldrich, Beijing). The minimum detection limits of such assays were as follows: 25 pg/mL for TNF-α and 10 pg/mL for IFN-γ, as previously determined by the manufacturer.

### Statistical analysis

Data were collected and analyzed using MS Excel 2010 and SPSS Statics v20.0 software. Weight loss, viral titers, cytokine levels and histological score were analyzed by analysis of variance (ANOVA), followed by Turkey’s multiple comparison test with *P* < 0.05 considered to be a significant difference, while *P* < 0.01 was considered to be statistically extremely significant.

## Results

### Virus titers

CIV was propagated in MDCK cells and the titers of three viral strains, JS/10, GD/12 and SD/05 were determined to be 10^7.13^ TCID_50_/mL, 10^7.25^ TCID_50_/mL and 10^8^ TCID_50_/mL, respectively.

### Characterization of mAbs

**Figure 1:**
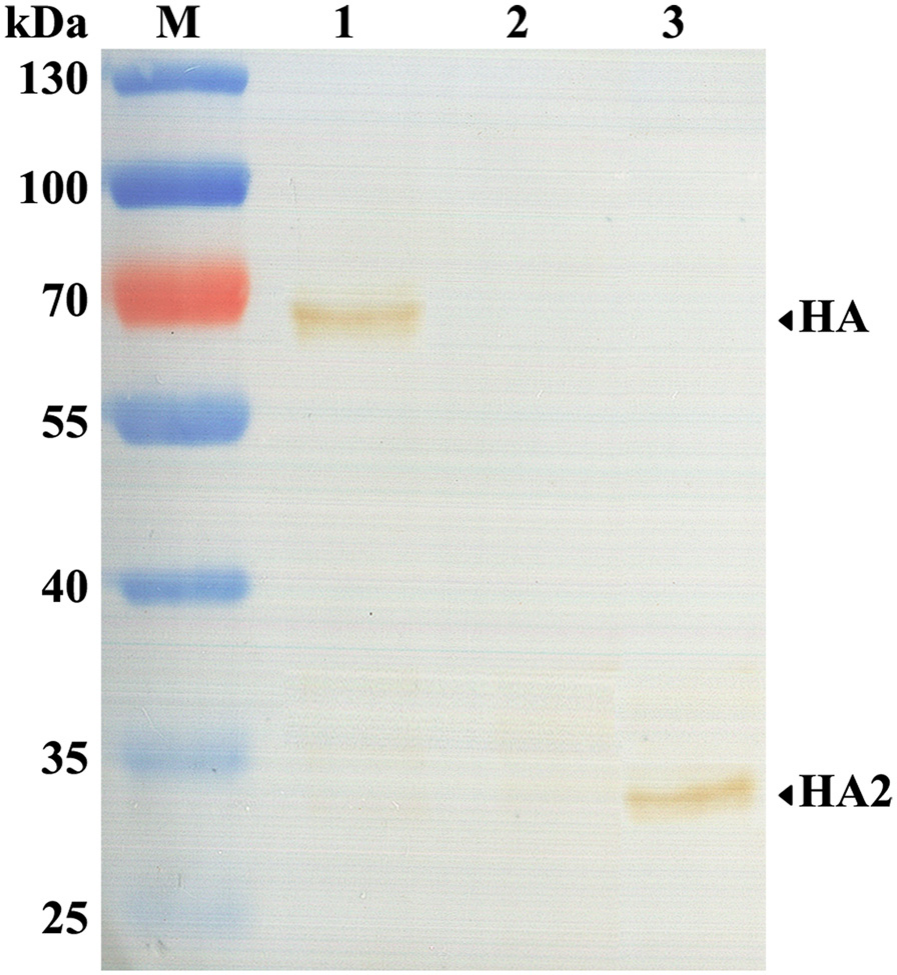
**Antigen identification of mAb D7 by western blotting using recombinant proteins HA, HA1 and HA2.** Lane M, protein pre-stained mass markers; Lane 1, mAb D7 reacted with expressed viral protein HA; Lane 2, mAb D7 reacted with recombinant protein HA1 (not visualized here); Lane 3, mAb D7 reacted with expressed protein HA2. Viral proteins were identified by DAB staining with HRP-labeled goat anti-mouse secondary antibody.

After fusion between spleen cells from H3N2 virus-immunized mice and sp2/0 myeloma cells, we obtained seven mAbs against the JS/10 virus. Isotyping tests showed that all of these mAbs were IgG2b isotypes, except for one that was IgG2a. Of the seven mAbs identified, four mAbs reacted with HA. Among these four mAbs, mAb D7 reacted with HA2 and three other mAbs reacted with HA1 (Figure 1), as demonstrated by western blotting. HI and neutralization titers of the seven mAbs showed that mAb D7 had the highest neutralization activity, but had no HI activity (Table 1). Further analysis indicated that mAb D7 could react with virus strains JS/10, GD/12 and SD/05, and produce high neutralization activities against the three viral strains, especially against the homologous strain JS/10 (Table 2).

**Table 1:** **Characteristics of seven monoclonal antibodies (mAbs) direct against JS/10**

**mAb**	**Isotype**	**Neutralization titer**	**HI titer**
B6	IgG2a	1600	320
B7	IgG2b	1600	80
B8	IgG2b	800	40
D7	IgG2b	12800	0
D8	IgG2b	6400	160
G6	IgG2b	400	20
H9	IgG2b	100	20

**Table 2:** **Characteristics of monoclonal antibody (mAb) D7 direct against virus strains JS/10, GD/12 and SD/05**

**Virus strains**	**mAb D7**		**mAb IgG**	
	**Neutralization titer**	**HI titer**	**Neutralization titer**	**HI titer**
JS/10	12800	0	0	0
GD/12	6400	0	0	0
SD/05	3200	0	0	0

### MAb D7 in the treatment of influenza in mice

To access the protective efficacy of mAb D7, we inoculated mice with three different H3N2 strains one day after treatment with mAb D7. Three days after the inoculation, all mice challenged with the three virus strains exhibited clinical signs of infection, including depression, decreased activity and huddling. Similar clinical signs were observed in irrelevant mAb IgG pretreated groups. However, similar to the PBS control group, mice in mAb D7 pretreated groups seemed to be energetic and had a good appetite during the infection.

**Figure 2:**
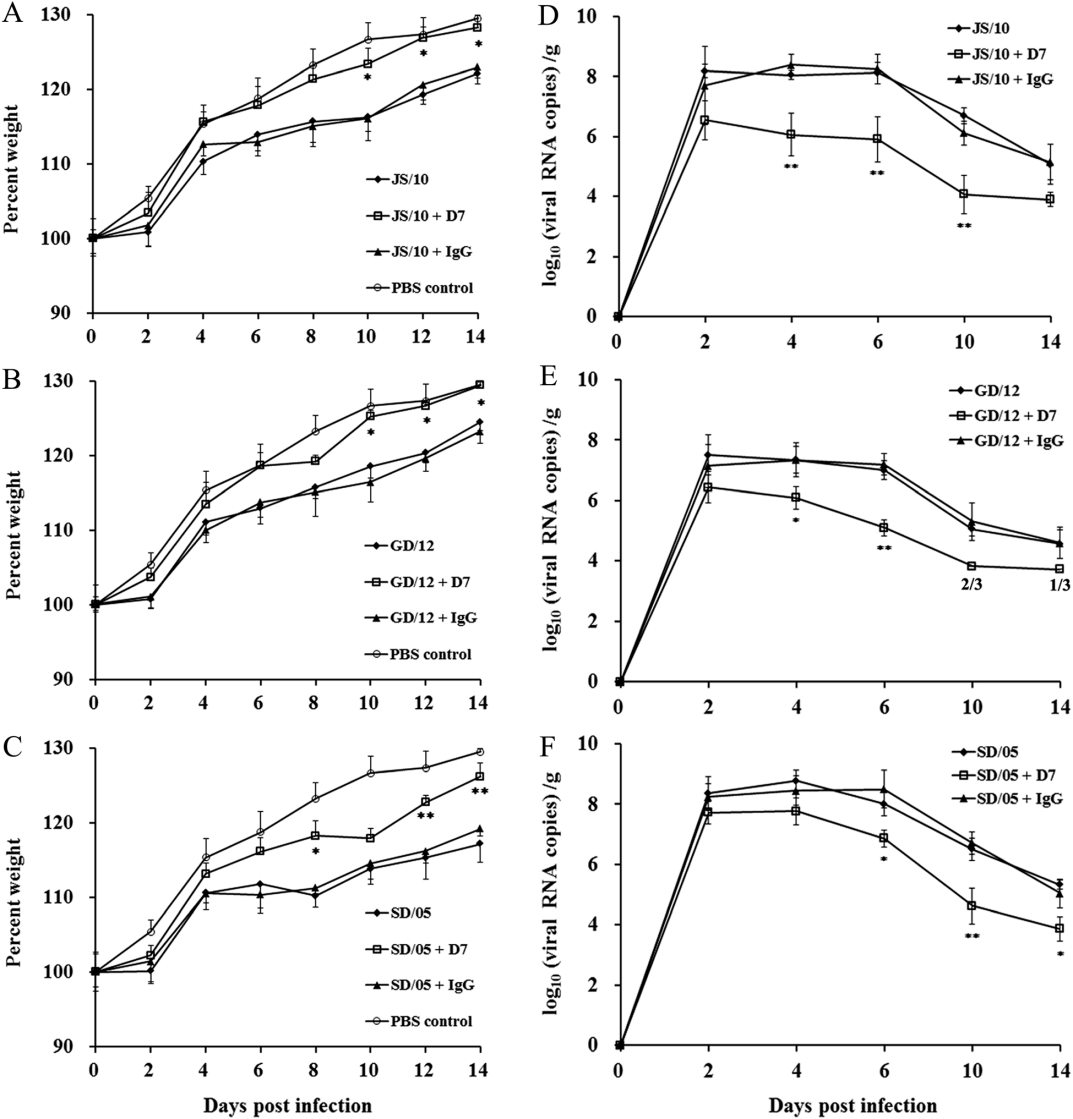
**Body weight changes and viral loads in the lungs of mice treated with mAb D7.** Three groups of 6-week-old BALB/c mice were challenged with approximately 10^7^ TCID_50_ of strains JS/10 **(A, D)**, GD/12 **(B, E)** and SD/05 **(C, F)**, respectively. In each virus group, mice were pretreated with 20 mg/kg of mAb D7, mAb IgG or PBS 1 day before viral challenge. Mice were monitored for body weight loss throughout the 14-day observation period. For body weight change **(A, B, C)**, the results are expressed in terms of percent body weight. *P < 0.05, or **P < 0.01, indicates a significant difference in weight data for the mAb D7 treated groups compared with the virus-infected groups and irrelevant mAb IgG groups. # *P* < 0.05, indicates a significant difference in weight data for the PBS group compared with the mAb D7 treated group. For viral loads in the lungs **(D, E, F)**, the results are expressed in terms of mean log10 number of copies/g of RNA standard deviation. *P < 0.05, or **P < 0.01, indicates a significantly different virus titer for the mAb D7 group compared with the other two groups. 2/3, and 1/3, indicate the proportion of the lungs in which virus could be detected.

In terms of body weight, mice challenged with JS/10 after treatment with D7 showed a similar increase in body weight compared with the PBS control group and there was no significant difference between the two groups (Figures 2A, B and C). At 14 dpi, mice treated with mAb D7 showed a body weight increase of nearly 30%. Although the body weights in the virus-infected group and irrelevant mAb IgG group both demonstrated an upward trend, the growth rate was slower than that in the mAb D7 group. The extent of the increase in body weight was significantly slower compared with that of the mAb D7 group at 10, 12 and 14 dpi (*P* < 0.05) (Figure 2A). In the group of mice infected with GD/12, the body weights of the mice in the three experimental groups all showed an upward trend, but the growth rate of the mice treated with mAb D7 was much higher than in the other two groups. In addition, the body weight changes of mice in the mAb D7 group at 10, 12 and 14 dpi were significantly different from those of the other two experimental groups (*P* < 0.05). The mice in the mAb D7 group showed weight gains of nearly 30%, which was not significantly different from the PBS control group (Figure 2B). However, after infection with SD/05, mice in the virus-infected group showed a slight decrease in body weight at 6 dpi and mice in the mAb IgG group displayed a slight decline at 8 dpi. By contrast, mice in the mAb D7 group continued to grow at 8 (*P* < 0.05), 12 (*P* < 0.01) and 14 dpi (*P* < 0.01). The growth rate in the mAb D7 group was significantly higher than that in the virus-infected group and mAb IgG group, but slightly lower than in the PBS control group at 14 dpi; mice body weight gain in the mAb group reached approximately 25% (Figure 2C).

### Quantitation of viral RNA loads

Real-time PCR was used to evaluate the kinetics of viral RNA loads in the lung, heart, brain, spleen, intestine, feces and blood of the infected mice. The viral titers were expressed as the number of copies of viral RNA. The dynamic changes of viral titers in the lungs of mice in the virus-infected group, mAb D7 group and irrelevant mAb IgG group were similar: peak viral titers were observed at 2, 4 and 6 days after infection, after which the viral titer declined at 10 and 14 dpi (Figures 2D, E and F). However, viral titers in the lungs of mice treated with mAb D7 were significantly lower than in the mice in the other two groups at specific time points. After challenge with JS/10, viral loads in lungs of mice treated with mAb D7 were significantly lower than those in the other two groups at 4, 6 and 10 dpi (*P* < 0.01) (Figure 2D). For strain GD/12, viral titers of the lungs in the mAb D7 group were significantly lower compared with the other two experimental groups at 4 (*P* < 0.05) and 6 dpi (*P* < 0.01). In addition, at 10 and 14 dpi, viral RNA could not be detected in some lung samples (1/3 and 2/3, respectively) in the mAb D7 group (Figure 2E). For virus SD/05, the viral titer of mice in the mAb D7 group was significantly lower than in the other two groups at 6 (*P* < 0.05), 10 (*P* < 0.01) and 14 dpi (*P* < 0.05) (Figure 2F). For mice in the virus-infected group, peak viral titers of three virus strains JS/10, GD/12 and SD/05 were 10^8.4^, 10^7.3^, 10^8.5^copies/g, respectively, while for mice in the mAb D7 group, peak values were 10^6.5^, 10^6.4^ and 10^7.8^ copies/g, respectively.

**Figure 3:**
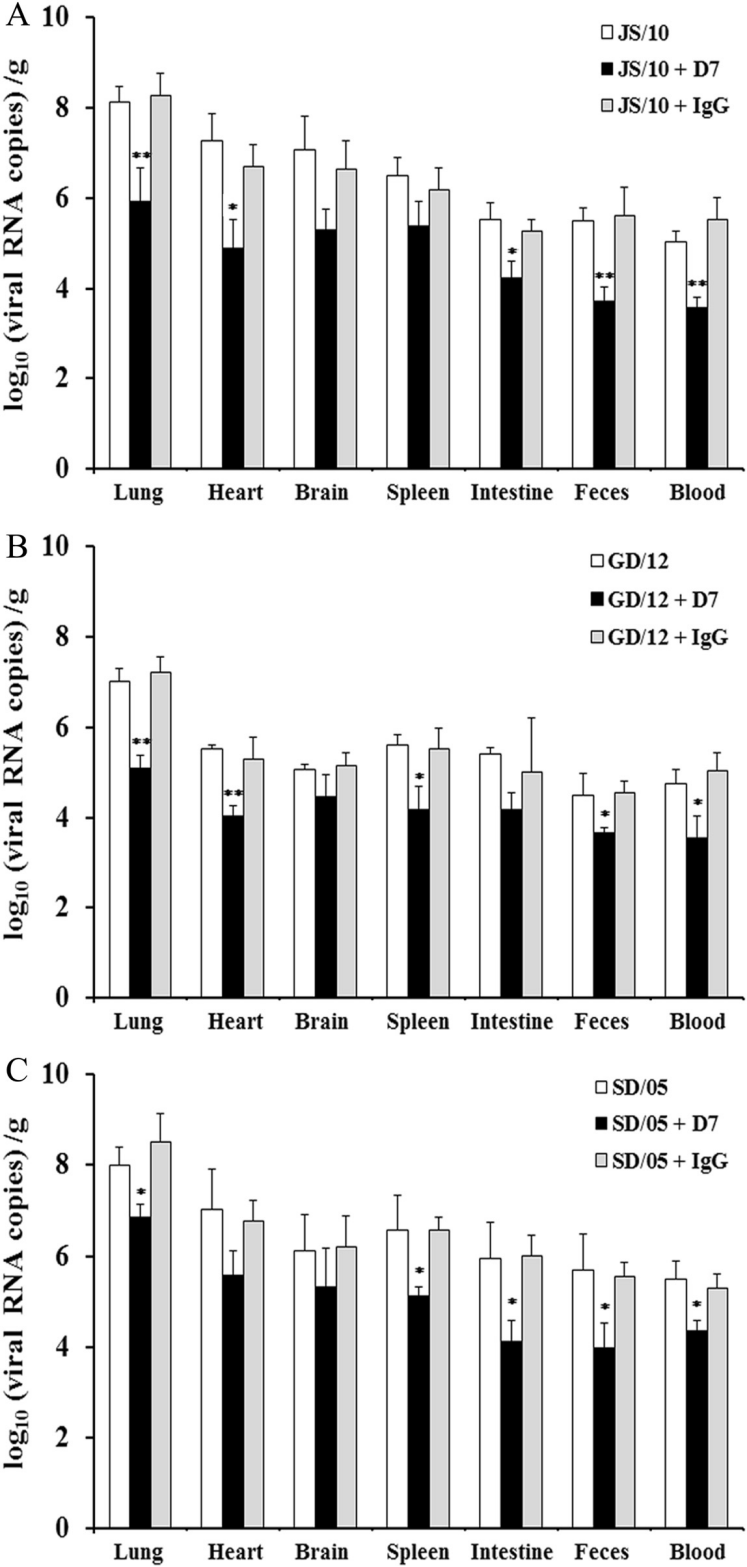
**Viral loads in collected tissues and fecal samples of mice at 6 dpi.** Mice were pretreated with 20 mg/kg of mAb D7, mAb IgG or PBS 1 day before viral challenge with virus JS/10 **(A)**, GD/12 **(B)** or SD/05 **(C)**, respectively. In each virus group, the lung, heart, brain, spleen, intestine, feces and blood of mice were collected for determination of viral loads using real-time PCR at 6 days post-challenge. *P < 0.05, or **P < 0.01, indicates significantly different virus titers compared with the other two groups.

Considering that mAb D7 resulted in a significant reduction in viral titers in the lungs of mice infected with three different virus strains at 6 dpi, we chose that time point to determine the viral RNA loads in different tissues and fecal samples. We found that viral loads in collected feces, blood and other tissues at 6 dpi in the mAb D7 group were also lower than those in the other two groups (Figure 3). After mice were challenged with virus JS/10, viral titers of the lung, heart, intestine, feces and blood in the mAb D7 group decreased by 192 (*P* < 0.01), 145 (*P* < 0.05), 20 (*P* < 0.05), 82 (*P* < 0.01) and 26 (*P* < 0.01) fold, respectively, compared to the other two groups (Figure 3A). For mice challenged with virus GD/12, viral titers of the lung, heart, spleen, feces and blood of mice treated with mAb D7 were found to be reduced by 103 (*P* < 0.01), 30 (*P* < 0.01), 25 (*P* < 0.05), 13 (*P* < 0.05) and 31 (*P* < 0.05) fold in comparison with the other two groups (Figure 3B). For virus SD/05, mAb D7 resulted in a reduction in viral titers of the lung, spleen, intestine, feces and blood by 13 (*P* < 0.05), 21 (*P* < 0.05), 57 (*P* < 0.05), 33 (*P* < 0.05) and 10 (*P* < 0.05) fold, respectively (Figure 3C).

Generally, mAb D7 could reduce viral loads of the three virus strains in the infected mice. Notably, in the virus-infected group and irrelevant mAb IgG group, after infection with virus JS/10, even till 14 dpi, virus RNA was detected in most tissues, while for the mice treated with mAb D7, virus RNA could not be detected in the brain and almost all the other tissues, except for the lung and blood, at 14 dpi (Additional file 1). For virus GD/12, at 10 dpi, viral titers in all the detected tissues were much lower compared with the other two viruses, in all three groups. Viral titers in some tissues were undetectable. Mice in the mAb D7 group showed a much faster virus clearance rate than the other two groups. At 10 dpi, no virus was detected in the intestines and feces of three mice in the mAb D7 group and at 14 dpi, virus was undetectable in all the other tissues in mice treated with mAb D7, except for the lungs in one mouse (Additional file 2). However, in mice infected with SD/05, the virus showed the longest retention time. At 14 dpi, nearly all tissues from mice in virus-infected and irrelevant mAb IgG groups showed detectable virus RNA. Even in the mAb D7 group, all mice showed positive virus RNA in the detected tissues, except for the intestines and feces (Additional file 3).

### Histopathological and immunohistochemical findings

**Figure 4:**
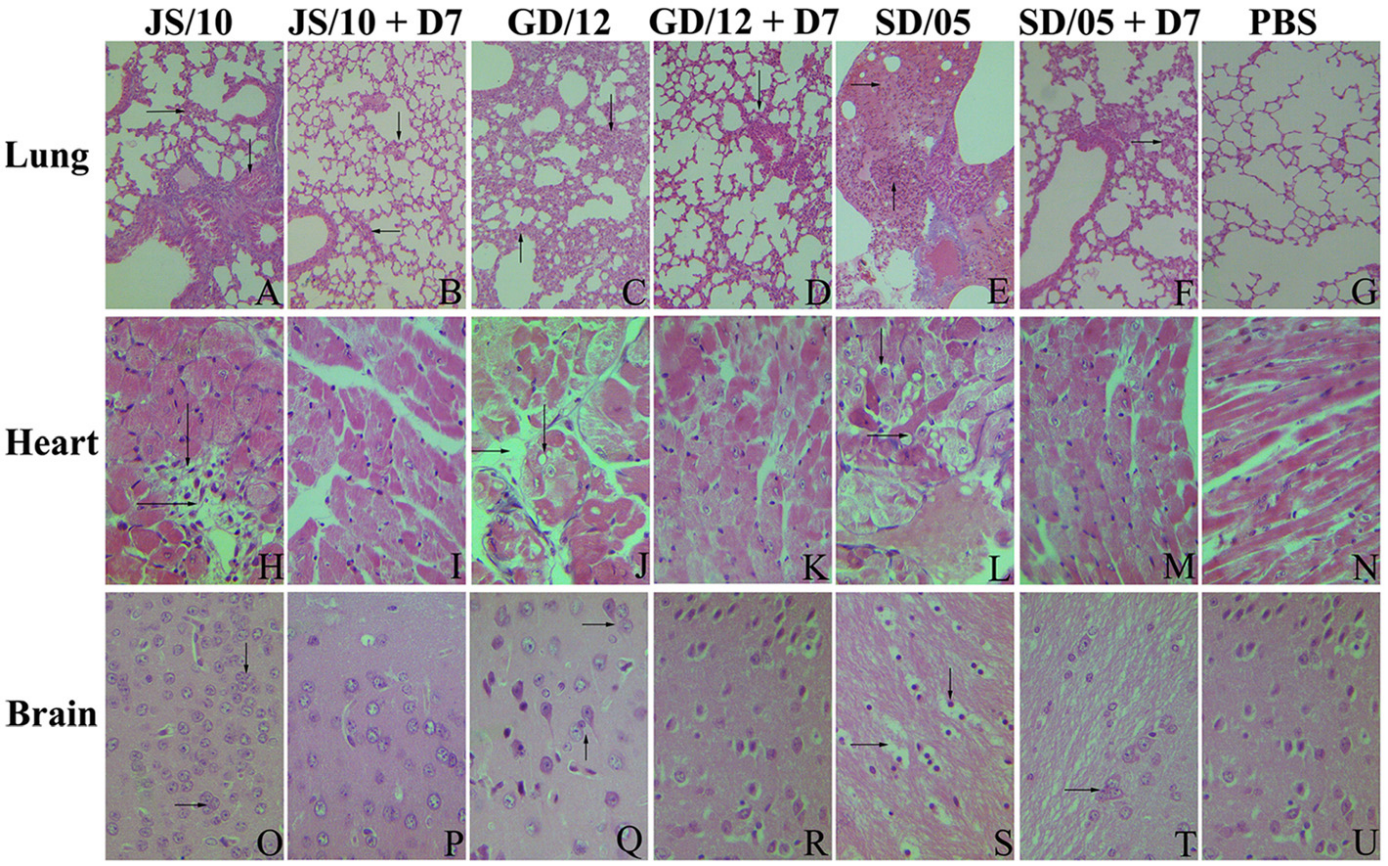
**Histopathological changes shown by H&E staining in the lung, heart and brain at 6 dpi.** For each virus, selected tissues from mice in the virus-infected group **(A, C, E, H, J, L, O, Q, S)**, the mAb D7 treated group **(B, D, F, I, K, M, P, R, T)** and the PBS group **(G, N, U)** are shown, respectively. Short arrows indicate the lesions. All the images are shown at 100 × magnification.

To compare the above results with pathological findings in mice infected with three different virus strains, and treated with mAb D7 and irrelevant mAb IgG, we chose the heart, brain and lung from different treatment groups at 6 dpi to perform histopathological and immunohistochemical analysis. All the sampled tissues from mice in the virus-infected group showed significant lesions and viral antigen staining, while those from the control group did not show any lesions. Mice treated with mAb D7 had markedly fewer lesions compared with the virus-infected group (Figure 4). Histological lesions in the mAb IgG group showed similar results with those in the virus-infected group (data not shown).

**Figure 5:**
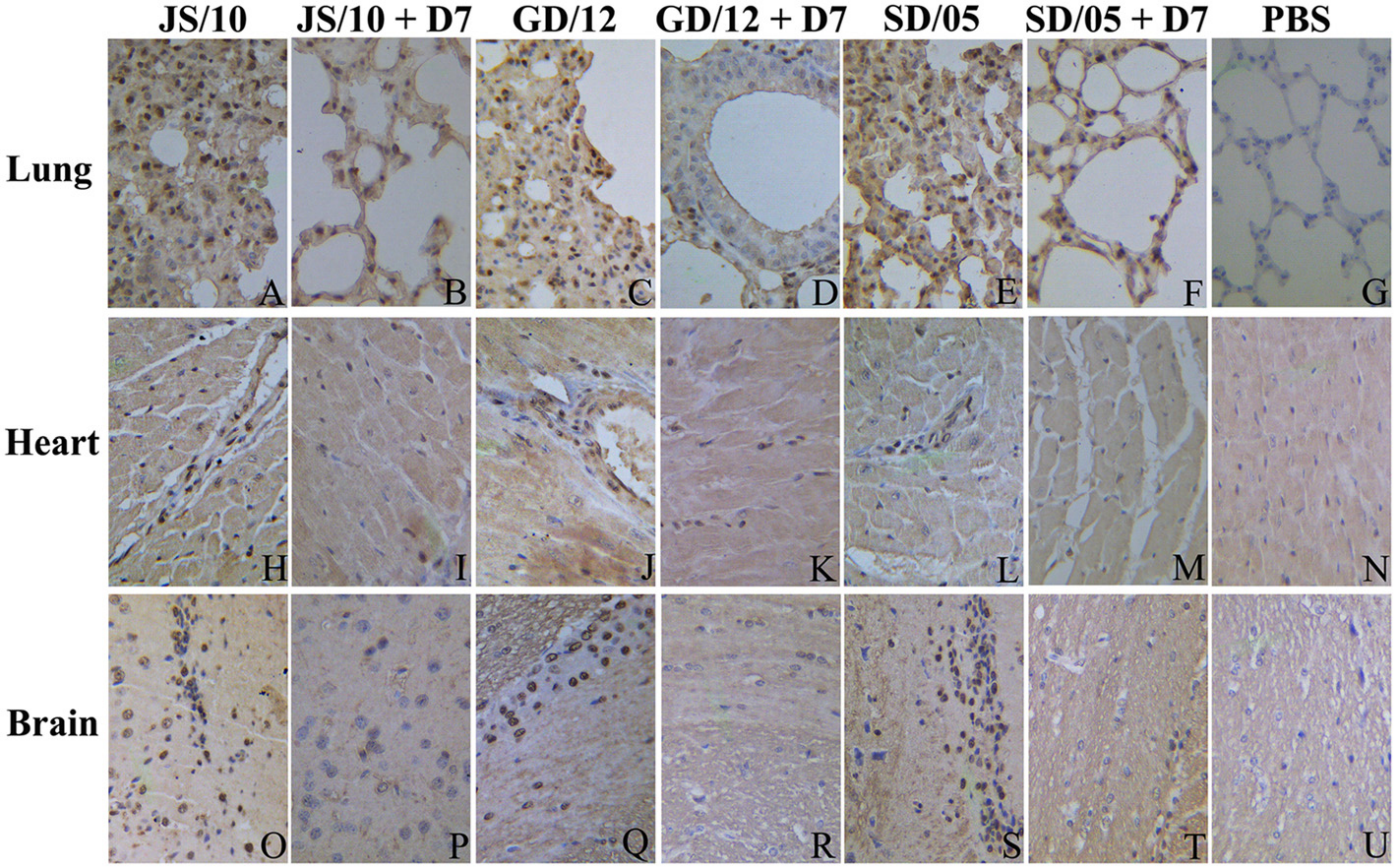
**Immunohistochemical detection of influenza viral antigen in the lung, heart and brain at 6 dpi.** For each virus, selected tissues from mice in the virus-infected group **(A, C, E, H, J, L, O, Q, S)**, the mAb D7 treated group **(B, D, F, I, K, M, P, R, T)** and the PBS group **(G, N, U)** are shown, respectively. All the images are shown at 400 × magnification.

**Figure 6:**
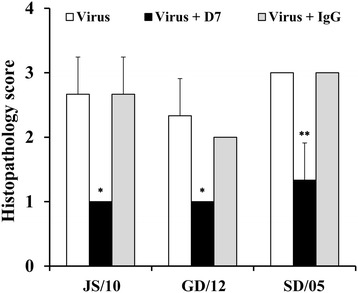
**The degree of lung injury after infection with virus JS/10, GD/12 and SD/05 at 6 dpi.** The lungs were assigned a grade 0 to 3 based on the histological character of the lesions. *P < 0.05, or **P < 0.01, indicates a significantly different score for the mAb D7 group compared with the other two groups.

Regardless of virus strain, the lung interstitial space was obviously widened, and the bronchial lumen became narrow, with the alveolar septum thickened by the infiltration of a number of inflammatory cells (Figures 4A and C). Large areas of the lung appeared consolidated, with symptoms of pulmonary congestion (Figure 4E). Interstitial pneumonia was also obvious, with the alveolar septum and proliferation of connective tissue infiltrated with numerous macrophages around the bronchioli and blood vessels (Figures 4C and E). In brief, histological lesions were characterized by multifocal to coalescing reddish consolidation in mice infected with the virus JS/10, GD/12 or SD/05 in both the virus-infected and irrelevant mAb IgG groups. However, the degree of histological lesions observed for SD/05 was the most severe, and GD/12 showed the least severe lesions among these three viruses. Mice treated with mAb D7 showed only mild necrotizing bronchiolitis and ciliated tracheal epithelium with mild hyperplasia (Figure 4F). In addition, very small gaps were observed between the alveoli and there were no excessive amounts of alveolar macrophages in the lung (Figures 4B and D). For virus strain GD/12, mAb D7 demonstrated the best protective efficacy compared with the other two viruses. Mice in the PBS group showed bronchia with a simple ciliated columnar epithelium and the alveolar cavity as a vacuolated thin-walled structure (Figure 4G). Viral antigen staining was present in almost all bronchiolar epithelial cells and some alveolar cells at 6 dpi after virus challenge (Figures 5A, C and E), while mice in mAb D7 showed only a little virus antigen staining surrounding vessels near the alveoli (Figures 5B, D and F). The PBS control group had no viral staining (Figure 5G). Lung grades for degree of injury at 6 dpi are shown in Figure 6. There was significantly less injury to the lungs in the mAb D7 group than in the virus-infected group and mAb IgG group for all virus strains.

Similar to the lung, all mice in the virus-infected groups showed histological lesions in the brain. The extent of histological lesions infected was the most severe with SD/05 and was the least severe for GD/12. The severity of the infection may depend on the differences in virus titer. In the cerebrum, congestion and hemorrhage were evident. Nerve fibers were dissolved and neurons had necrolysis-like vacuoles; glial nodules and neuronophagia were also observed (Figures 4O and Q). Dilation and hyperemia were found in the capillaries (Figure 4S). Moreover, microglial cells and nerve cells showed a satellite phenomenon. The cytoplasm of the neurons was basophilic because of contraction (Figures 4O and Q). Viral antigens could be detected in glial nodules and microglial-gathered areas (Figures 5O, Q, and S). The brains of mice treated with mAb D7 had only mild lesions surrounding microglial cells and nerve cells (Figures 4P and T), and almost no antigen staining was found (Figures 5R and T). The PBS control group showed no histological findings (Figure 4U) and no viral staining (Figure 5U).

In the heart, for all mice in virus-infected groups, the cardiac striated muscle was disordered and full of vacuoles, characterized by myocarditis, and lymphocyte infiltration was observed (Figure 4H). Lymphoproliferation was also found among the muscle fibers (Figure 4J). The nuclei showed pyknosis, with some myocardial cells showing coagulative necrosis (Figures 4J and L), which was consistent with heavy antigen staining (Figures 5H, J and L). No significant differences in microscopic lesions (Figures 4I, K, M and N) and viral antigen staining (Figures 5I, K, M and N) were found between the mAb D7 and PBS groups.

### Cytokine response in lung tissue

To gain a better understanding of the effect of CIV on the innate immune response and to ascertain whether passive immunization with monoclonal antibody affected the levels of cytokines, we examined the levels of IFN-γ and TNF-α in the lungs of mice in the virus-infected and mAb D7 groups.

**Figure 7:**
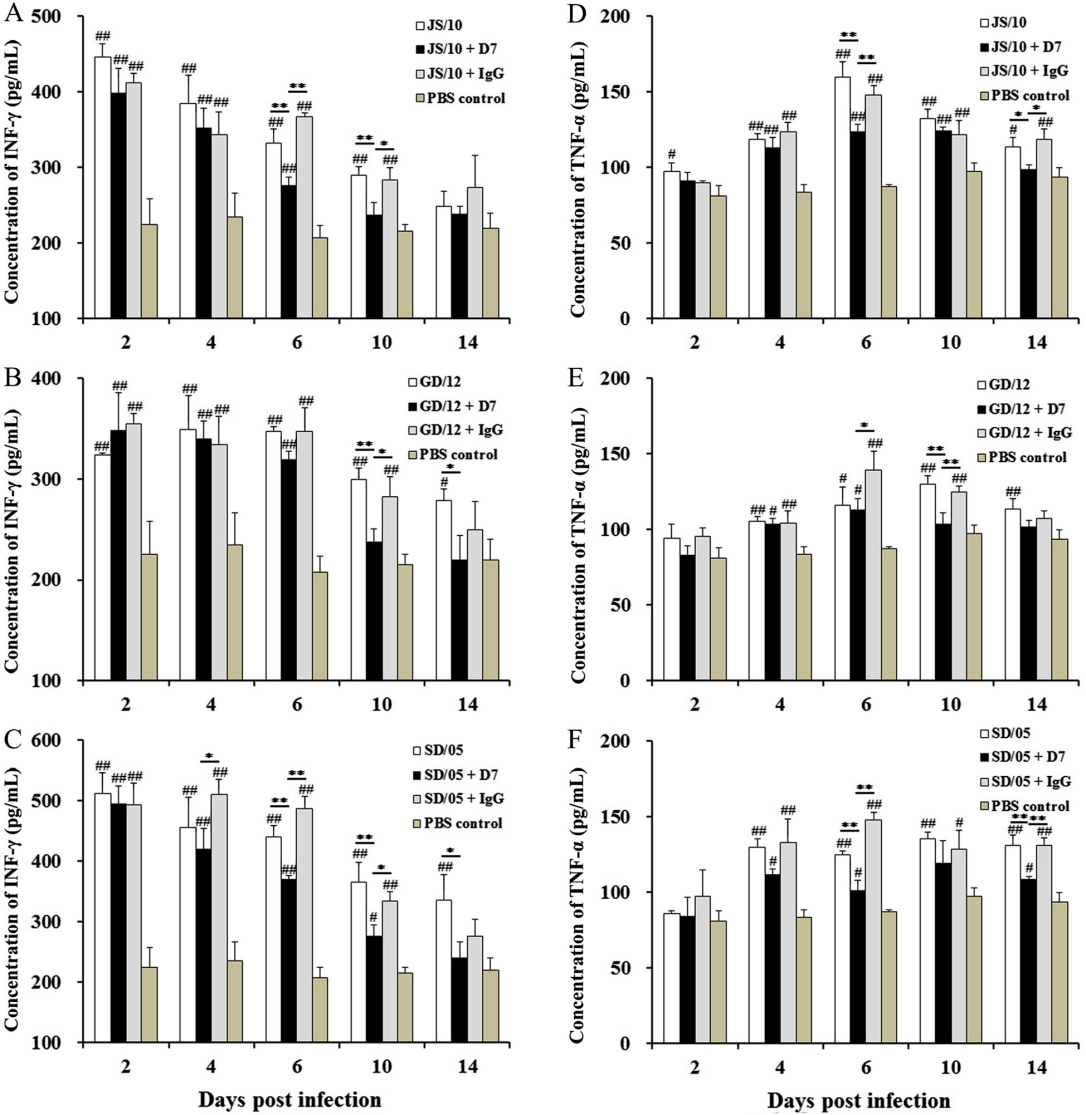
**Characterization of IFN-γ and TNF-α secretion from lung tissues of mice challenged with virus.** Cytokine concentrations were measured by ELISA in supernatants of homogenates from the lungs infected with three virus strains. Mice were pretreated with 20 mg/kg of mAb D7, mAb IgG or PBS 1 day before challenged with virus JS/10 **(A, D)**, GD/12 **(B, E)** or SD/05 **(C, F)**, respectively. The cytokine levels were measured in the infected mice on days 2, 4, 6, 10 and 14 post challenged. The results are expressed in terms of pg/mL. *P < 0.05, or **P < 0.01 indicates significantly different changes for mAb D7 group compared with the virus-infected group or the mAb IgG group. # *P* < 0.05, or ## *P* < 0.01, indicates a significant difference between this group and the PBS control group.

As shown in Figure 7, the level of IFN-γ in response to all three virus strains showed an identical trend, higher at 2, 4 and 6 dpi than at 10 and 14 dpi. The IFN-γ levels in mAb D7 group in all three virus strains were significantly lower than the virus-infected group or mAb IgG group, especially at 6, 10 and 14 dpi. However, the cytokine levels differed with the various virus strains.

For virus strain JS/10, the IFN-γ level of all groups reached its peak at 2 dpi, and then exhibited an overall downward trend. The cytokine levels in the mAb D7 group at 6 (*P* < 0.01) and 10 dpi (*P* < 0.05) were significantly lower than that in the virus-infected or mAb IgG group; however, the IFN-γ level did not show any significant difference among the three groups at other time points (Figure 7A). The IFN-γ level in the mAb D7 group returned to normal after 10 dpi, while the other two groups returned to the normal level at 14 dpi.

For virus strain GD/12, the IFN-γ levels of the three experimental groups were significantly higher than in the mice in the PBS group at 2 (*P* < 0.01), 4 (*P* < 0.01) and 6 dpi (*P* < 0.01), sustaining a relatively high level until 10 dpi, after which it decreased. The peak level of IFN-γ in the mAb IgG group was lower compared with the other two virus strains at 2 dpi (Figure 7B). Although the IFN-γ levels in the three experimental groups challenged with GD/12 did not show much difference compared with each other, the IFN-γ level in the mAb D7 group at 10 dpi returned to a normal level, which was significantly lower (*P* < 0.05) compared with the other two groups; all the three groups showed normal levels at 14 dpi.

For virus strain SD/05, this virus induced the largest rise in IFN-γ levels. The level in the SD/05-infected group was significantly higher than that of the other two viruses at 2 dpi. In addition, mice in virus-infected and mAb IgG groups both demonstrated the same trend in the period of 2 to 10 dpi (*P* < 0.01), i.e., significant elevation followed by a downward trend (Figure 7C). Although the IFN-γ level in the mAb D7 group at 2 to 10 dpi was relatively high compared with the PBS group, the IFN-γ levels were significantly lower than those of the other two groups at 6 (*P* < 0.01) and 10 dpi (*P* < 0.05), and then declined to the normal level at 14 dpi.

Changes in TNF-α level were not the same as those for IFN-γ. After infection with the three virus strains, TNF-α levels of mice in all groups were only slightly higher than those of the PBS group at 2 dpi and increased at 4 and 6 dpi. Levels reached their maximum at 10 dpi and decreased at 14 dpi, however, the cytokine level was still higher than that of the control group at 14 dpi which was quite different compared with the IFN-γ level. The increase in TNF-α level was lower compared with the IFN-γ level. In addition, the TNF-α level in the mAb D7 group was apparently lower than the virus-infected group or mAb IgG group, especially at 10 and 14 dpi.

For virus strain JS/10, TNF-α levels in the virus-infected group and mAb IgG group showed a small rise at 2 dpi, reaching its peak at 6 dpi, and then declined; however, the cytokine level was still significantly higher (*P* < 0.05) than that of the PBS group (Figure 7D). The TNF-α level in the mAb D7 group at 14 dpi (*P* < 0.05) was remarkably lower than that in the virus-infected group and mAb IgG group, and decreased to a similar level as the PBS group.

For virus strain GD/12, the TNF-α level of all three experimental groups increased from 2 to 6 dpi, and then returned to normal at 14 dpi (Figure 7E). The TNF-α level of the mAb D7 group at 10 dpi was significantly lower (*P* < 0.05) than in the other two groups, while the cytokine level did not show much difference in these three groups at the other time points.

For virus strain SD/05, the TNF-α levels in the virus-infected group and mAb D7 group were markedly higher than those of the PBS group from 4 to 14 dpi (Figure 7F). Although the TNF-α level in the mAb D7 group was also significantly higher (*P* < 0.05) than the PBS group, except at 2 and 10 dpi, the level in the mAb D7 group was significantly lower (*P* < 0.01) compared with the virus-infected and irrelevant mAb IgG groups at 6 and 14 dpi.

## Discussion

H3N2 CIV is a newly identified avian influenza virus (AIV) subtype that can infect dogs and transmit directly from dog to dog [5,8]. CIV infection has been reported in several countries, including South Korea and China [9,10,37]. It is important to develop a set of measures to prevent and control CIV infection in dogs.

Antibody-mediated passive immunity can provide protection against invading pathogens [38,39]. In this study, we developed seven mAbs against JS/10, whose pathogenicity has been characterized both in mice [8] and dogs [40]. Among them, four mAbs reacted with HA. The HA glycoprotein is the primary target of antibodies that confer protective immunity to influenza viruses [41]. Therefore, the generation of neutralizing antibodies against antigenic sites on the HA glycoprotein is regarded as a criterion for evaluating immunity to influenza viruses and is believed to constitute the main correlate of protection [42,43]. Anti-HA globular head mAbs have potent neutralizing activity against homologous strains, but have very limited breadth of reactivity because of the high variability of amino-acid changes in the HA1 globular head [44]. HA2, which is the HA stalk, however, is a conserved region of HA among all influenza A virus subtypes [33,45] and is responsible for the fusion of the virus and the endosomal membrane during the entry of the virus into the cell [20]. Here, western blotting showed that MAb D7 recognized the HA2 domain of H3, and had highest neutralization activities. Although MAb D7 lacked HI activity, some previous studies reported that a lack of in vitro HI activity of anti-HA2 MAbs does not rule out protective activity in vivo [33,45]. Therefore, we selected the anti-HA2 MAb D7 for further evaluation in regards to protection against different influenza virus strains.

To investigate the protection of mAb D7 against homologous and heterologous strains of H3N2 influenza viruses, we selected three virus strains to perform the challenge experiment in mice, including two strains of CIV (JS/10 and GD/12) and one strain of swine influenza virus (SIV) (SD/05). Considering that almost all H3N2 CIV isolates reported were not lethal to mice or dogs in challenge experiment [5,8,10,15,40], we evaluated the protection efficacy by body weights, viral loads and histological lesions. Body weight loss is the parameter most commonly used to evaluate influenza viral pathogenicity in mice [46]. Our study shows that all three virus strains could remarkably reduce the growth rate of the mice after infection, while pretreatment with mAb D7 helped to control the declination to some extent. From the pathological point of view, the lung, heart and brain in mAb D7 treated groups showed markedly fewer lesions compared with the virus-infected group and mAb IgG group, with all virus strains. The pathological scores of the lungs in mAb D7 group were lower than those in the virus-infected group and mAb IgG group, suggesting that mAb D7 could mitigate the damage caused by influenza virus. These results suggest that mAb D7 could offer a protective effect against the three virus strains.

To further evaluate the effects of the anti-influenza virus mAb, we monitored viral loads by real-time PCR. The mAb D7 decreased the viral loads in the lungs to significantly lower levels, relative to those in the virus-infected group and mAb IgG group. Similar results were also found in other tissues. For JS/10, the virus in the brain and other organs of all three mice treated with mAb D7 had been cleared by 10 dpi, except for the lungs. For virus GD/12, the application of mAb D7 caused virus clearance from the intestine and feces 4 days earlier than that in the other two groups; moreover, there was no detectable virus RNA at 14 dpi. For virus SD/05, a slower rate of clearance of viral load was observed. This virus could persist and be detected in most tissues in the mAb D7 group until 14 dpi, but virus in the intestine and feces had been cleared by 14 dpi. These results indicate that protection against the virus strains provided by mAb D7 might be caused by earlier clearance of the virus from the tissues or shortening the time of virus shedding. A previous study has reported that mAbs could reduce the period of virus clearance [23].

Our study indicates that mAb D7 could provide good protection against challenge with homologous, as well as heterologous, virus strains of H3N2 influenza virus. This finding was in accordance with a previous report [47] that HA2 mAbs are highly cross-reactive among strains of the same subtype, and even within different subtypes. In spite of this, we found that this mAb was relatively less effective against the swine-lineage than canine-lineage H3 virus strains. Sequence analysis of amino acids showed that JS/10 had higher sequence identity to canine-lineage GD/12 (98.6%) than to swine-lineage SD/05 (84.8%), with 3 and 16 different amino acids, respectively, in HA2 (data not shown). The difference in amino acid sequence may affect antigen-antibody recognition. This may explain why the mAb induced by canine-lineage influenza virus strain JS/10 is not strong enough to react against more distantly related strains within the same subtype.

Cytokines are important in establishing an innate immune response, as well as in determining the magnitude of the inflammatory response to influenza virus infection. The most important feature of the mechanism of immune suppression with influenza virus H5N1 is the cytokine storm [11,48]. Here, to gain a better understanding of how virus infection and mAb treatment affected host immune response, we analyzed the levels of IFN-γ and TNF-α in the lung.

An important finding in this study is that the TNF-α levels peaked two days later than IFN-γ levels in all groups. A previous study showed similar results: IFN-γ and TNF-α in the lungs of pigs infected with human H1N1 influenza virus peaked at 3 dpi, and 6 dpi respectively [49]. IFN-γ has important immune-regulatory functions and antiviral activity, and is primarily produced by natural killer (NK) and T cells. NK cells are a major player in innate immune responses. We speculated that early production of IFN-γ during infection probably arises from NK cells, whereas TNF-α functions relatively late in the inflammatory cycle induced by infection, at a time when virus is already being contained and the response is centered on resolution of the inflammation [50,51]. TNF-α in mice challenged by JS/10 and SD/05 maintained higher levels until 14 dpi. A previous study demonstrated that the depletion of TNF-α in influenza or respiratory syncytial virus-infected animals significantly reduced pulmonary inflammation and cytokine production, without compromising viral clearance [52]. We speculated that the elevated level of TNF-α at 14 dpi might be correlated with the uncleared virus loads in the lungs.

In the present study, three strains of H3N2 CIV were shown to induce elevated levels of cytokines in the lungs. This was in agreement with previous reports on H3N2 CIV [37,53]. During H5N1 influenza virus infection, the elevated pro-inflammatory cytokine response has been proposed as the main cause of the increased severity of the disease [54]. The study of Lee et al. [55] reported that the levels of IFN-γ and TNF-α in the lungs of dogs infected with H3N2 influenza virus increased quickly, while the infected dogs developed severe bronchointerstitial pneumonia accompanied with massive infiltration of immune cells. This result suggests that the dysregulation of chemokines during H3N2 CIV infection might contribute to viral pneumonia characterized by extensive immune cell infiltration. In support of this hypothesis, we observed that elevated levels of cytokines accompanied the clinical manifestations in the CIV infected dogs.

We found that IFN-γ and TNF-α levels in the mAb D7 group were significantly lower than in the virus-infected group or mAb IgG group, while the histopathological findings showed more significant lesions in lungs of mice from the latter two groups than in the mAb D7 group. These observations indicate that mAb D7 treatment may reduce the virus-induced cytokine production and pathological lesions caused by virus infection. The effect is likely to be mediated by inhibition of CIV replication by the mAb. Fritz et al. [50] reported that active influenza virus replication is required for the induction of potent proinflammatory, regulatory and chemotactic factors.

Our study and a previous report [55] demonstrate that active replication of CIV in the canine respiratory system results in intense inflammatory responses. Considering that it is important for the host to maintain a balance of the cytokine levels, we speculated that inhibition of the inflammatory cytokine response might offer a therapy for CIV infection. However, Salomon et al. [56] demonstrated that inhibition of the cytokine response during H5N1 influenza virus infection is not sufficient to protect against death, and proposed that therapies targeting the virus would be preferable.

In conclusion, our results suggest that the HA2-specific mAb D7 could contribute to early recovery from influenza infection with different H3N2 virus strains. This mAb will further our understanding of the antigenic properties of H3N2 virus and might contribute to the prevention and control of H3N2 virus epidemic in dogs.

## Additional files

Additional file 1:
**Viral RNA detection in collected samples of mice inoculated with virus strain JS/10.** Numbers 1, 2 and 3 represent the three mice euthanized from each group at different dpi. + represents viral loads in terms of mean log10 number of copies/g of RNA. Numbers of +’s represent the magnitude of RNA copies. Additional file 2:
**Viral RNA detection in collected samples of mice inoculated with virus strain GD/12.** Numbers 1, 2 and 3 represent the three mice euthanized from each group at different dpi. + represents viral loads in terms of mean log10 number of copies/g of RNA. Numbers of +’s represent the magnitude of RNA copies. Additional file 3:
**Viral RNA detection in collected samples of mice inoculated with virus strain SD/05.** Numbers 1, 2 and 3 represent the three mice euthanized from each group at different dpi. + represents viral loads in terms of mean log10 number of copies/g of RNA. Numbers of +’s represent the magnitude of RNA copies.
