# Supplementary material for: Monoclonal antibody specific to HA2 glycopeptide protects mice from H3N2 influenza virus infection
Source: Vet Res. 2015 Mar 19;46(1):33. doi: 10.1186/s13567-015-0146-7 (PMC4364502; doi:10.1186/s13567-015-0146-7)
Supplement: Additional file 2: — Viral RNA detection in collected samples of mice inoculated with virus strain GD/12. Numbers 1, 2 and 3 represent the three mice euthanized from each group at different dpi. + represents viral loads in terms of mean log10 number of copies/g of RNA. Numbers of +’s represent the magnitude of RNA copies. [file 13567_2015_146_MOESM2_ESM.doc]

**Additional file 2 Viral RNA detection in collected samples of mice inoculated with virus strain GD/12.**

| **Group** | **Organ** |  | **2 dpi** | | |  | **4 dpi** | | |  | **6 dpi** | | |  | **10 dpi** | | |  | **14 dpi** | | |
| --- | --- | --- | --- | --- | --- | --- | --- | --- | --- | --- | --- | --- | --- | --- | --- | --- | --- | --- | --- | --- | --- |
|  |  |  | **1** | **2** | **3** |  | **1** | **2** | **3** |  | **1** | **2** | **3** |  | **1** | **2** | **3** |  | **1** | **2** | **3** |
|  | **Lung** |  | **++++++++** | **+++++++** | **++++++++** |  | **+++++++** | **+++++++** | **++++++++** |  | **+++++++** | **+++++++** | **+++++++** |  | **+++++** | **+++++** | **+++++** |  | **+++++** | **+++++** | **++++** |
|  | **Heart** |  | **+++++++** | **++++++** | **+++++++** |  | **+++++++** | **+++++++** | **+++++++** |  | **++++++** | **+++++** | **+++++** |  | **++++** | **++++** | **+++++** |  | **++++** | **++++** | **+++++** |
|  | **Brain** |  | **+++++++** | **+++++++** | **++++++** |  | **+++++** | **+++++** | **++++++** |  | **+++++** | **+++++** | **+++++** |  | **-** | **+++++** | **+++++** |  | **-** | **++++** | **-** |
| **GD/12** | **Spleen** |  | **+++++++** | **+++++++** | **+++++++** |  | **++++++** | **++++++** | **+++++++** |  | **+++++** | **++++++** | **+++++** |  | **+++++** | **++++** | **+++++** |  | **++++** | **++++** | **-** |
|  | **Intestine** |  | **+++++++** | **+++++++** | **+++++++** |  | **++++++** | **++++++** | **++++++** |  | **+++++** | **+++++** | **++++++** |  | **+++++** | **+++** | **-** |  | **-** | **++++** | **-** |
|  | **Feces** |  | **+++++++** | **++++++** | **++++++** |  | **+++++** | **+++++** | **++++++** |  | **++++** | **+++++** | **++++** |  | **-** | **+++++** | **-** |  | **-** | **-** | **-** |
|  | **Blood** |  | **++++++** | **++++++** | **++++++** |  | **+++++** | **++++++** | **+++++** |  | **+++++** | **++++** | **+++++** |  | **++++** | **+++++** | **++++** |  | **++++** | **++++** | **++++** |
|  | **Lung** |  | **++++++** | **+++++++** | **+++++++** |  | **++++++** | **++++++** | **+++++++** |  | **+++++** | **+++++** | **+++++** |  | **-** | **+++** | **++++** |  | **-** | **-** | **++++** |
|  | **Heart** |  | **+++++** | **+++++** | **++++++** |  | **++++++** | **++++++** | **+++++** |  | **++++** | **++++** | **++++** |  | **-** | **++++** | **-** |  | **-** | **-** | **-** |
|  | **Brain** |  | **+++++** | **+++++** | **+++++** |  | **++++** | **+++++** | **++++** |  | **++++** | **+++++** | **++++** |  | **-** | **-** | **++++** |  | **-** | **-** | **-** |
| **GD/12 + D7** | **Spleen** |  | **+++++** | **++++++** | **+++++** |  | **++++++** | **+++++** | **++++++** |  | **+++++** | **++++** | **++++** |  | **-** | **-** | **++++** |  | **-** | **-** | **-** |
|  | **Intestine** |  | **+++++** | **+++++** | **++++++** |  | **+++++** | **+++++** | **+++++** |  | **++++** | **++++** | **+++++** |  | **-** | **-** | **-** |  | **-** | **-** | **-** |
|  | **Feces** |  | **++++++** | **+++++** | **+++++** |  | **+++++** | **++++** | **+++++** |  | **++++** | **++++** | **++++** |  | **-** | **-** | **-** |  | **-** | **-** | **-** |
|  | **Blood** |  | **+++++** | **+++++** | **+++++** |  | **+++++** | **+++++** | **+++++** |  | **+++** | **+++** | **++++** |  | **-** | **++++** | **++++** |  | **-** | **-** | **-** |
|  | **Lung** |  | **+++++++** | **++++++++** | **++++++** |  | **++++++++** | **+++++++** | **++++++++** |  | **++++++++** | **+++++++** | **+++++++** |  | **+++++** | **+++++** | **++++++** |  | **++++** | **++++** | **+++++** |
|  | **Heart** |  | **+++++++** | **+++++++** | **++++++** |  | **+++++++** | **++++++** | **+++++++** |  | **+++++** | **+++++** | **++++++** |  | **++++** | **++++** | **+++++** |  | **++++** | **-** | **++++** |
|  | **Brain** |  | **++++++** | **++++++** | **++++++** |  | **++++++** | **++++++** | **++++++** |  | **+++++** | **+++++** | **+++++** |  | **++++** | **-** | **+++++** |  | **++++** | **-** | **-** |
| **GD/12 + IgG** | **Spleen** |  | **++++++** | **++++++++** | **+++++++** |  | **++++++** | **+++++++** | **+++++++** |  | **+++++** | **++++++** | **++++++** |  | **+++++** | **+++++** | **+++++** |  | **+++++** | **++++** | **-** |
|  | **Intestine** |  | **++++++** | **+++++++** | **++++++** |  | **+++++++** | **++++++** | **++++++** |  | **+++++** | **+++++** | **++++** |  | **++++** | **-** | **+++++** |  | **++++** | **+++++** | **-** |
|  | **Feces** |  | **+++++++** | **++++++** | **+++++** |  | **++++++** | **+++++** | **+++++** |  | **++++** | **++++** | **+++** |  | - | **-** | **+++++** |  | **++++** | **-** | **-** |
|  | **Blood** |  | **++++++** | **++++++** | **++++++** |  | **+++++** | **+++++** | **++++++** |  | **+++++** | **++++++** | **+++++** |  | **++++** | **+++++** | **++++** |  | **+++++** | **++++** | **-** |

Numbers 1, 2 and 3 represent the three mice euthanized from each group at different dpi. + represents viral loads in terms of mean log10 number of copies/g of RNA. Numbers of +’s represent the magnitude of RNA copies.
